# Land Use/Cover Change Detection and Urban Sprawl Analysis in Bandar Abbas City, Iran

**DOI:** 10.1155/2014/690872

**Published:** 2014-09-02

**Authors:** Mohsen Dadras, Helmi Zulhaidi Mohd Shafri, Noordin Ahmad, Biswajeet Pradhan, Sahabeh Safarpour

**Affiliations:** ^1^Department of Civil Engineering, Faculty of Engineering, Universiti Putra Malaysia (UPM), Serdang, 43400 Selangor, Malaysia; ^2^Department of Civil Engineering, Faculty of Engineering, Islamic Azad University, Bandar Abbas, Iran; ^3^Geospatial Information Science Research Centre (GISRC), Faculty of Engineering, Universiti Putra Malaysia, 43400 Serdang, Selangor, Malaysia; ^4^School of Physics, Universiti Sains Malaysia, 11800 Penang, Malaysia

## Abstract

The process of land use change and urban sprawl has been considered as a prominent characteristic of urban development. This study aims to investigate urban growth process in Bandar Abbas city, Iran, focusing on urban sprawl and land use change during 1956–2012. To calculate urban sprawl and land use changes, aerial photos and satellite images are utilized in different time spans. The results demonstrate that urban region area has changed from 403.77 to 4959.59 hectares between 1956 and 2012. Moreover, the population has increased more than 30 times in last six decades. The major part of population growth is related to migration from other parts the country to Bandar Abbas city. Considering the speed of urban sprawl growth rate, the scale and the role of the city have changed from medium and regional to large scale and transregional. Due to natural and structural limitations, more than 80% of barren lands, stone cliffs, beach zone, and agricultural lands are occupied by built-up areas. Our results revealed that the irregular expansion of Bandar Abbas city must be controlled so that sustainable development could be achieved.

## 1. Introduction

UN projections show that population settled in urban regions in 2006 was 50% of whole population of the world and it would be almost 60% by 2020 [1]. The major part of this growth belongs to developing countries. Although increasing growth of cities in south hemisphere (countries such as, China, India, and Brazil) is a well-known phenomenon, formation, change of the form, and procedure of urban growth are still unknown phenomena [2–5]. Urban growth in most countries includes widening process and expanding pattern; however, procedures such as expansion and sprawl are just a part of urban growth procedure [6–8]. Factors such as density of buildings, elevation of buildings, and development plans play a prominent role in urban growth [9, 10]. Urbanization has vital influence both on the abiotic environment [11] and the living organisms of the cities [12].

Land use change generates new patterns of urban sprawl. Urban sprawl is divided into eight separate dimensions including concentration, density, continuity, proximity, centrality, clustering, nuclearity, and mixed uses [13]. Concentration is a degree of development which is inappropriately located in an area less than 1 square mile from whole city area. The process of urban growth in these circumstances is completely asymmetrical and sprawl. In urban growth field, density is determined in three forms: building density (floor area ratio (FAR), which is the buildings floor area to the size of piece of land where it is built), net residential density (number of dwellings divided by residential land area in that region), and gross residential density (population of a region divided by area of the whole land of that region). Developable lands, where construction is performed, located in the proximity of urban fabric zone are determined by continuity measure. Proximity is a measure which defines various land uses which are near each other all over the urban region. Centrality is a measure specifying residential and nonresidential developments (both of them) which are close to central business district (CBD) of an urban region. Clustering is a measure of development demonstrating the minimum land area in each mile which is capable of being developed and occupied by residential and nonresidential land uses. Nuclearity is the value which defines expansion limit of an urban region using single core development pattern (in contrast with multicore pattern). Finally, mixed use is a sign of specifying different land uses to one small area [13]. Considering the aforementioned characteristics, unbalanced urban sprawl is a serious threat for sustainable urban development. Thus, urban expansion models such as smart growth are presented which try to damp and inverse increasing trend of destructive environmental effects using eight aforementioned dimensions [14–16].

Urban growth studies in most aspects concentrate on big cities and metropolises. Nevertheless, medium and small urban regions may possess maximum urban growth rate in a specific time interval from their establishment time. For instance, Weng [17] in his study on Zhujiang delta in China figured out that the maximum urban growth happened in Nanhui, Bao'an, Dongguan, and Zhuhai regions which are relatively small cities located on the eastern part of the delta, whereas no trend of urban lands increment was observed in older and larger cities such as Guangzhou and Foshan. Jat et al. [18] investigated increase of urban lands in Ajmer city as a medium city in Rajasthan state of India. The obtained results revealed that population of the city had become more than threefold during the past 25 years. The urban region area has changed from 488 hectares in 1997 to 1259 hectares in 2002. Sudhira et al. [19] conducted a study on cities with population less than 5 million people in India. They reported that population has increased 54% between 1972 and 1999. According to their report, at the same time, urban regions increased 146% which is almost three times of population growth rate. Land sprawl has been studied in medium and small cities in developed countries, for example, California and Santa Barbara [20] or some cities in Switzerland [21] and lots of other samples are investigated.

Despite large amount of studies performed on urban sprawl in developed countries, few studies have been conducted on reasons and influential factors affecting urban sprawl in developing world. In this paper, both urban formation and development were assessed using aerial photos and satellite images. Remote sensing is known as an economical tool and efficient technology; therefore, it could be vastly utilized to monitor and analyze urban sprawl process [22, 23]. Furthermore, remote sensing provides particular advantages with respect to describing spatial and local trends of urban sprawl [24, 25]. Thus, the possibility of checking changes of area and urban land uses in the past as well as planning urbanization process in the future is achieved using images from different time intervals. The goal of this study is to employ remote sensing data and GIS techniques to investigate and analyze urban growth procedure and land use changes in Bandar Abbas city during the last 57 years.

In recent years Bandar Abbas city has encountered accelerated and wide spread urban growth due to its strategic location, proximity to main ports of import and export, and existence of several industrial zones near it. According to first performed census in Iran in 1956, the number of Iran's cities was 201 and the ratio of urban population to whole population was 29%. In 2012 the number of cities became 1331 and the ratio of urban population became 70% of whole population [26, 27]. UN projections demonstrate that Iranian urban population will reach to 80% of whole population by 2020. Large cities such as Isfahan, Mashhad, and Tehran are experiencing transfer of urban growth procedure from compressed form to sprawl form [28]. In some medium and small cities this procedure is rapidly expanding dependent on their location and specific characteristics. Bandar Abbas city is a large city whose physical growth and land cover/use change have been rapid during recent years. Since definitions of counties and urban hierarchies are diverse in different countries, in this study urban sprawl and process of land use/cover change in Bandar Abbas city are investigated based on population and exploiting urban hierarchies in Iran.

## 2. Materials and Methods

### 2.1. Study Area

**Figure 1:**
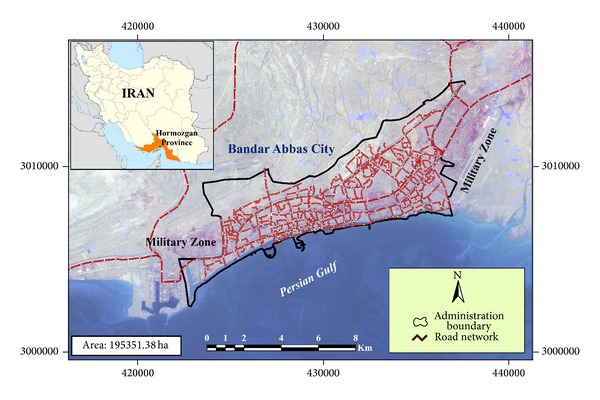
Bandar Abbas location in Iran.

Bandar Abbas city is located in South of Iran and beside the Persian Gulf and it is capital of Hormozgan province (Figure 1). It is one of the most important southern ports of Iran and it used to be named Bandar Gambroun. It is located in a hot and humid region. Summer continues almost nine months in this city. The temperature fluctuates between 44 and 2 Celsius degrees during the hottest and coldest day of the year, respectively. The average precipitation in Bandar Abbas is almost 200 mm. Bandar Abbas is surrounded by mountains and high altitude regions from North and sea from South. Hence, general slope of the city is from North to South. A considerable area of the city including Souris district in Southwest of the city, southern side of Imam Khomeini street between Shilat Khor and Gour Souzan Khor and South of Nakhl-e-Nakhoda district are smooth land with altitude between 0.6 and 5 meters higher than sea level. Starting Iran-Iraq war while prominent commercial ports of Iran such as Khoramshahr and Abadan were destroyed, Shahid Rajaee commercial port in Bandar Abbas became the main and most strategic commercial port of Iran. In the beginning of 90s, when the war finished, economic growth of Iran increased drastically. It changed form −1.2 percent in 1975 to 2.5 percent in 1990. The aforementioned factors as well as establishment of refinery, steel, and aluminum industries increased migration of job seeking population to Bandar Abbas. Tourist attraction is another functionality of this city as it has several commercial centers, it is seaside, and it consists of coral islands and lots of natural landscapes. Annually more than 5 million people visit Bandar Abbas and its nearby islands. The mentioned factors have brought up accelerated and spread growth to Bandar Abbas in comparison with other cities of the country. According to census of Iranian Statistics Center [27] the population of Bandar Abbas was about 520000 in 2012. Considering 2.3% growth rate, it will be 820000 by 2030. Thus, Bandar Abbas will be considered as one of the large cities of Iran. According to standards determined by Iranian Statistics Center [27, 29] and Ministry of Roads and Urban Development [30, 31], Iran's cities are classified into six levels considering size and population. These levels include megalopolis (population is more than 5 million people), metropolis (population between 1 and 5 million), large city (population between 500000 and 1000000), medium city (population 100000 and 500000), small city (population between 25000 and 100000), and rural-urban (population less than 25000). So Bandar Abbas is categorized as a large city with 520000 people. At the beginning, the focus of this study was the formation of Bandar Abbas. Investigating characteristics of urban growth and land use/cover changes in medium and large cities of developing world is efficient and beneficial.

### 2.2. Land Use/Cover Change

Detecting land use/cover change (LULC) provides a proper background for environmental, planning, and urban management analysis. However, detecting land use/cover is not easy due to various factors. Comparative analysis using classification operation enables us to detect trend of land use/cover changes in different times. The utilized methods include map-to-map comparison and postclassification comparison. There might be two essential problems on our way to do this stage. First, the exact boundaries of polygons might not be distinguished properly due to lack of precision. In vector formats this problem is known as “slivers” which can be defined as narrow polygons of dubious interpretation [24, 28, 32, 33]. These slivers clogged up the system and clouded analysis. For these technical reasons, most early work on integration of map sources used a grid cell approach. In raster formats slivers cause the presence of border pixels with false positive or negative changes. False positive changes occur when a change is identified but no change has taken place and false negative changes appear when no change is identified but a change has taken place. Second probable problem occurs when the size of pixels in land cover/use map is different. In this case some elements will not be recognized properly due to lack of resolution or lack of precision in detachment of their locations [32]. In this study, remote sensing data was different so both problems existed. In order to analyze land use/cover change, nine periods of remote sensing data were utilized. Among them there were six main periods (aerial photos: 1956, 1965, 1975, 1987, and 2001; GeoEye-1 satellite image in 2012) and three auxiliary ones (Aerial photo Ultra Cam-d 2013 and satellite images: Landsat 2003 and IRS 2008) (Table 1). The time intervals which were considered for detecting urban land use/cover changes depend on time intervals of remote sensing data and are almost 10 years. First of all, mosaicking, orthorectification, and triangulation of aerial photos were performed through photogrammetry using Erdas Imaging 2011 software. It should be mentioned that coordinates of 1300 points were taken using bifrequency Trimble DGPS device. These points are utilized for georeferencing, error adjustment, and geometric corrections. The photogrammetry operation performed on aerial photos taken by Ultra CAM-d camera was different from analog photos (the difference is because of its digital structure and including GPS and IMU technologies; these two technologies facilitate geometric correction based on external justification using (*X*, *Y*, *Z*, *ω*, *φ*, *κ*) parameters). Subsequent to performing photogrammetry on aerial photos based on stereoscopy (using stereoscope and 3D binoculars), terrains were interpreted and detected. Afterwards, polygons detected in aerial photos were extracted to classify land cover/use units of each year. Transforming generated polygons to vector format using ESRI-ArcGIS 10 software, types of land use/cover, and their area were determined.

After atmospheric, radiometric, and geometric correction of satellite images used by Erdas Imaging 2011 software, the classification process was performed (supervised and unsupervised). In this process, terrains and types of their use were detected. The results of the classification accuracy for satellite images are presented in Table 2. In the next step boundaries were set for detected terrains using polygons which surround locations with detected use. Then, the generated polygons were imported to analyzing environment of Geographic Information System (ESRI-Arc GIS 10 software). GIS tools and technologies were exploited to assign land uses, estimate area, and analyze land use/covers detected in different time intervals. As a matter of fact when all satellite images and aerial photos are provided, land use/cover changes might be detected via two different approaches: the first, automatic classification and the second, interpretation of images and photos. Classification units of remote sensing data regarding land use/cover changes consist of 8 classes including (1) agriculture lands, (2) barren lands, (3) coastal zone, (4) marsh lands, (5) military zones, (6) river, (7) cliff hills, and (8) urban lands and built-up areas. Built-up areas (impervious) are generally considered as a quantitative parameter which describes urban expansion [34–37]. Class of built-up land use includes residential areas consisting of single houses and apartments, trade centers, industrial and commercial facilities, highways and main roads, parking lots, urban green space, and their characteristics. Agricultural lands of studied region consist of farms, gardens, shrimp ponds, and livestock. Sandy deserts, sand dunes, and unutilized lands constitute barren lands. Coastal zones refer to regions where tides occur and are located in the southern part of the city. There are marsh lands in the western part of Bandar Abbas city which have resulted from slope and subsidence. These areas have been changed to marsh lands during the time. Military zones, which are considered as one of the most crucial constraints imposed on urban physical development, have been placed in eastern, central, and western parts of the city during the time and by various governments. It is noticeable that from another viewpoint military functionalities are taken into account as an important priority affecting urban crisis management and urban security. Rivers of Bandar Abbas are seasonal; they are flooding in rainy seasons and they are dry in arid seasons. Frontages of seasonal rivers in the region have changed due to climate circumstances and violation caused by urban construction. Unfortunately, enough attention is not paid to frontage of seasonal rivers and safety considerations; as a result, in rainy seasons, river flooding occurs destroying a large part of urban lands resulting in financial loss and casualties. Major part of northern lands of studied region includes cliffs due to geological formation. Cliff hills are considered as natural limitations of urban expansion.

**Figure 2:**
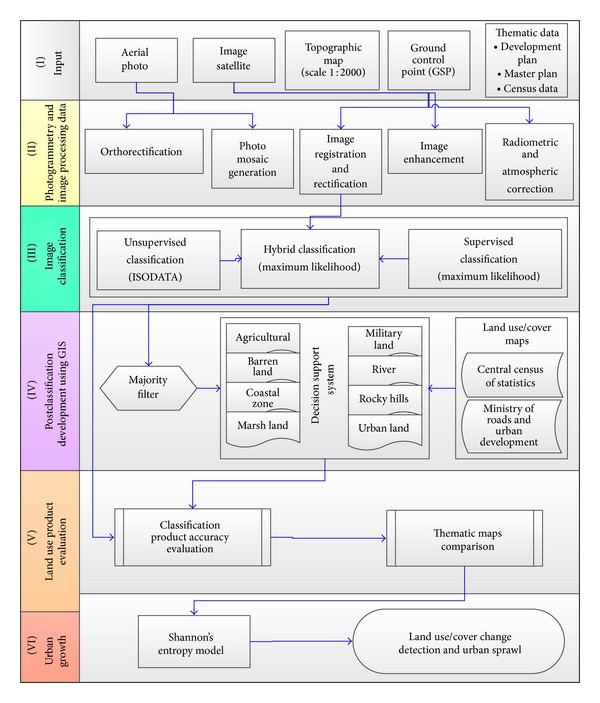
Methodology of detecting trend of land use/cover changes (LULC) in Bandar Abbas city.

Since it is difficult to distinguish some land use/cover through automatic classification, computer interpretation of aerial photos and satellite images as well as field observations was utilized [38, 39]. In this method interpretation and classification of remote sensing data is followed by field observation so that achieved results could be compared and upgraded. Visual interpretation is the best approach which helps us to avoid generating lots of incorrect classifications. This scheme is taken into account as the best approach to obtaining correct information regarding procedure of land use changes [40]. Furthermore, this method facilitates generalizing and distinguishing between uses of various lands, detecting trend of changes, formation pattern, and characteristics related to each land use [33]. To interpret aerial photos and satellite images in desired region, firstly, aerial photo taken in 1956 was prepared applying photogrammetry operation so that land use/cover could be interpreted and detected. After classification and extraction (Erdas Imaging 2011 software), detected terrains are transformed to vector format (ESRI-Arc GIS 10). Then, results achieved from interpretation and classification of 1956 aerial photo are overlaid with those of 1965 aerial photo. Subsequently, the procedure of interpretation and detection of land use/cover is conducted based on upgrading results obtained from previous period (1956) in order to provide land use/cover map of 1965. The same procedure is employed for years 1975, 1987, 2001, 2003, 2008, 2012, and 2013. It must be noticed that field observations performed in 2013 at 1300 points aimed to check the results yielded through interpretation and classification of land use/cover.  Figure 2 depicts the procedure of analysis and extraction of results for land use changes.

## 3. Results

**Figure 3:**
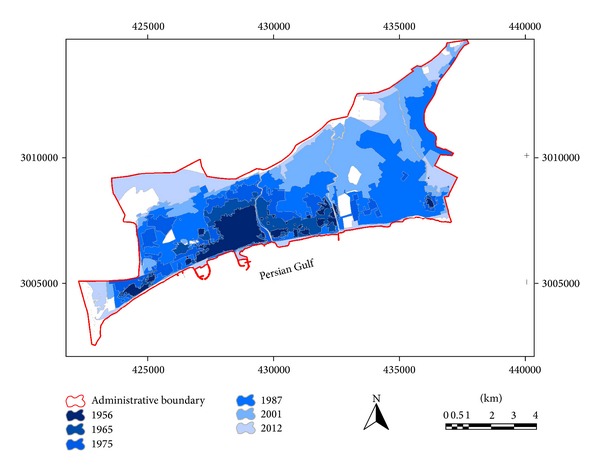
Urban growth and expansion of Bandar Abbas city (1956–2012).

Urban planning in Iran and Bandar Abbas shows several challenges of urban growth. Structure of urban planning in Iran is formed based on transregional scale development plans, master plan, and detailed plan. The first master plan for Bandar Abbas city was prepared in 1967 by Dr. Adibi Consultant Company. In this plan 2.3% population growth with a population density equaling to 100 people in each hectare was predicted. According to prediction of 1967 master plan, the area of Bandar Abbas city was supposed to reach 1250 in 1987. As illustrated in Table 3 the area of built-up regions equals 2914.64 hectares which is almost twice of the predicted amount (Figure 3). Political, social, and economic processes played an important role in excessive growth of Bandar Abbas city during the 20-year time span (1967–1987). The second master plan for Bandar Abbas was provided in 1984. As there were unutilized spaces between built-up regions, the urban growth was predicted in these regions. Furthermore, excessive growth of city boundaries and development of fringing areas were restricted in mentioned master plan. In other words, in this plan urban growth in empty spaces between urban lands was considered as a priority. Yet, urban boundaries grew toward North and Northeast due to lack of supervision and improper implementation of issues predicted in master plan. The last master plan for Bandar Abbas city was prepared by Sharmand Consultant Company in 2005. Unfortunately, the plan suffers from several shortcomings and it has not been passed by council of architecture and urban development. Despite natural and structural constraints of physical urban development, the area of Bandar Abbas city is predicted to be 10325 hectares in 2020.

To sum up, the main reasons which have avoided realization of predictions performed by master plans in Bandar Abbas city include excessive migration to the city, growth of fringing area, lack of reconstruction in old regions of the city, imperfect execution of master plans, and lack of supervision on administrative process. Also, political, economic, and social evolutions have played an important role in the growth of Bandar Abbas city in recent decades.

### 3.1. Population Growth

**Figure 4:**
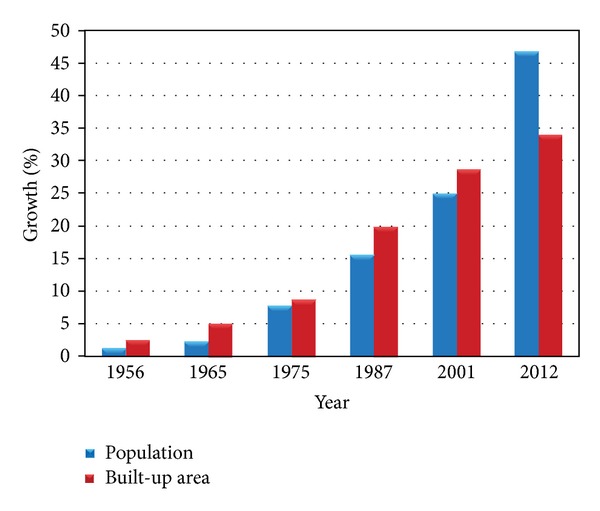
Population growth to area of built-up area ratio in Bandar Abbas city (1956–2012).

According to the first official census performed in Bandar Abbas by ministry of internal affairs in 1956, the population of Bandar Abbas was 17710 people [31, 41]. As mentioned earlier, during 1965–1970 times span land development and other economic plans in Iran encouraged a great number of people to migrate to urban areas. As a result in the second official census (1975) the population of Bandar Abbas was 87981. After 1979 Islamic revolution and as a consequence of Iran-Iraq war, considerable number of people migrated from war affected cities to other cities. Bandar Abbas city hosted the major part of war affected migrants due to economic growth and urban development. The annual population growth rate in this city was 5.8% between 1975 and 1987. The population became 276578 in 2001 with annual population growth rate equal to 5.5%. As officially reported through census performed in 2012 the population of Bandar Abbas was 518345 (Figure 4).

**Figure 5:**
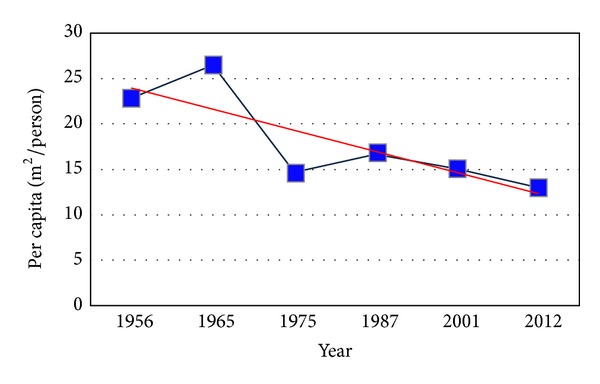
Per capita changes of built-up areas during 1956–2012.

As can be seen in Figure 5 and Table 3, rate of population growth is extremely higher than urban lands and built-up area growth. Equation (1) shows significance relationship between rates of population change and built-up area growth as follows:

(1) $$\begin{array}{c} Y=-2.3277x+26.272,\\ R^{2}=0.668. \end{array}$$

### 3.2. Urban Sprawl

**Figure 6:**
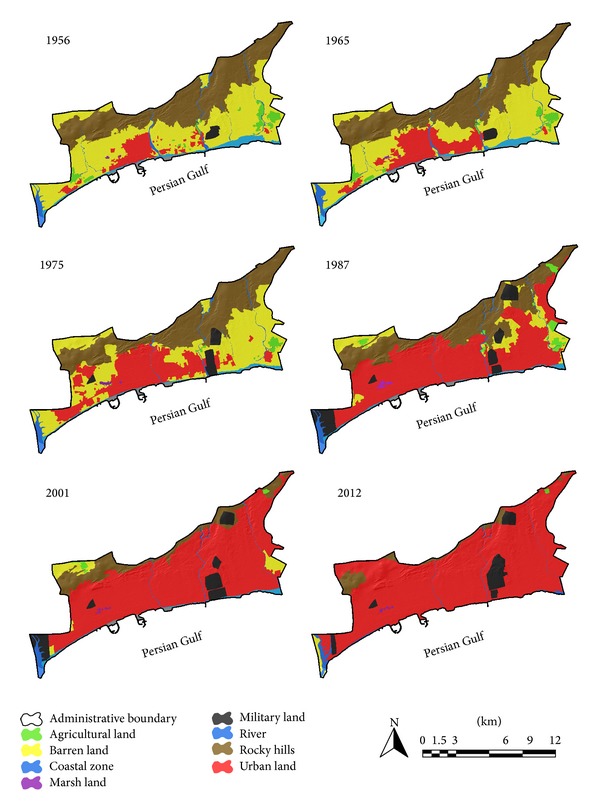
Land use changes maps (1956–2012).

To estimate land use changes and urban expansion trends aerial photos of 1956, 1965, 1975, 1987, and 2001 as well as satellite image of 2012 were utilized as main data. Furthermore, aerial photos of 2003, 2008, and 2013 were exploited as secondary data. The procedure of land use changes in urban region of Bandar Abbas during six time spans is shown in Table 3. Analyzing aerial photos of 1956 demonstrates that urban regions area and built-up areas were 403.77 hectares. Population density in this year was almost 43.86 people per hectare. As illustrated in Figure 3, physical growth and development of Bandar Abbas city basically occurred in the proximity of primary core of the city (its location is Southwestern part of the city) and expanded toward different directions later. This process of urban growth has changed considerably during the time. In Figure 3 it is evident that urban region in 1965 had mostly expanded toward North, East, and Southeast and the area of the city had become 753.78. The same trend was followed and urban region of Bandar Abbas city grew 125.45% and became 2914.64 during 1975–1987. It means that the urban region had become twice its area in previous time span (aerial photo 1975). Additionally, the population increased 98.51% in the same period of time (Table 2). During 1987–2001 built-up areas increased and urban empty spaces were filled. Urban lands show a considerable growth in 2001 which was almost 43.51% (4182.88 hectares) (Table 3 and Figure 3). In the last time period (2001–2012) the major part of empty urban spaces was filled as a result of increase of construction density. In 2012 Bandar Abbas urban region was 4959.59 hectares and the population density was 77.27 people per hectare. This irregular expansion demonstrates chaos and abnormality in construction and population density in Bandar Abbas during six past decades. On the other hand, location of built-up areas and population density are not appropriate in most parts of Bandar Abbas (Figure 6).

Built-up areas in Bandar Abbas have increased from 403.77 hectares in 1956 to 4959.59 hectares in 2012. Achieved results presented in Table 3 show that population growth had a larger value comparing to land development rate. During 1956–2012 built-up areas increased 354.73% whereas population increased 514.26% (Figure 4 and Table 3). Several factors in different time periods influenced urban population growth. During 10 recent years, establishment of factories and huge industries such as steel and aluminum in addition to development of Bandar Abbas refinery and Shahid Rajaee harbor has resulted in great migration toward Bandar Abbas. As shown in Table 3 during 2001–2012 population amount was roughly multiplied by two. Also according to Table 4 per capita land consumption has increased drastically during the last three decades. Land consumption per capita indeed shows usage of land capabilities for developing urban uses such as official, educational, commercial, industrial, residential, and amusement.

### 3.3. Land Use Transformations

**Figure 7:**
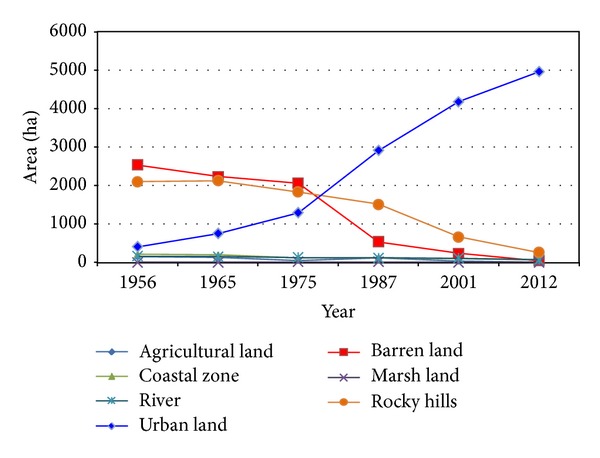
Land use changes in Bandar Abbas city (1956–2012).

The results of land use changes in Bandar Abbas during six past decades are shown in Figure 6 and Table 5. The most frequent change was transforming barren lands to built-up areas (urban use). So, almost 2489 hectares of barren lands were assigned to urban uses (official, commercial, educational, cultural, sport, and so on). Another group of major changes have occurred in cliff hills, agricultural zones, and coastal zone. These zones have been occupied with urban uses. During recent years, a large portion of coastal and agricultural zones has been manipulated by urban construction due to lack of supervision and demerit of officials. In Bandar Abbas city a large part of beach is occupied by beach road connecting Southeast to Southwest. Although coastal and agricultural zones are tourist attractions and environmental savings of the city, they have drastically decreased during recent years. Coastal zones decreased from 210.96 hectares in 1956 to 60.31 hectares in 2012. On the other hand, area of agricultural zones is reduced from 155.62 hectares in 1956 to 6.92 hectares in 2012. As it is clearly seen in Figures 1 and 6 Bandar Abbas city cannot be developed so much as a result of natural (high altitude regions in North, coastal zone in South) and structural (air force and airport in East and navy in the West) limitations. This fact has caused lands located in the North of the city (which are mostly covered by cliffs) to be occupied by urban constructions. The area of rugged land (cliffs) which are connected to urban region was 2097.24 hectares in 1956 and it is reduced to 262.16 hectares in 2012 (Table 4). Land shortage and lack of proper management of urban lands have resulted in use of rugged lands which are not suitable for urban use and construction. Table 5 demonstrates the matrix of land use/cover changes in 8 levels for specified land uses in Bandar Abbas city between 1956 and 2012. This table shows that urban and built-up lands increased by 15 times as much (4508.43 hectares). This was a decreasing trend in barren lands with −2489.14 hectares, coastal zone which was −150.66 hectares, and agricultural lands which were −148.70 hectares. The obtained results show that the major part of land uses is occupied by built-up areas. According to data achieved from classification maps and land use change matrix, it might be concluded that urban expansion has caused widespread land use changes in Bandar Abbas city. The land use map in master plan prepared in 2005 demonstrates that almost 74% of urban region is dedicated to built-up lands and urban use and almost 26% of remained lands are empty an unutilized [30]. The maximum increasing changes in area of land use belong to urban lands while the most decreasing area belongs to barren and cliff areas (Figure 7).

As it can be seen in Figure 6 that formation of Bandar Abbas city is linear. During the time and considering urban sprawl, urban formation and growth have experienced great changes. Natural restrictions in North and South as well as structural limitations in West and East have caused high performance lands to be occupied by urban uses. The process of land use change in Bandar Abbas city during last 57 years is illustrated in Figure 7. Most of changes belong to urban lands and they had incremental trend. The urban land growth has caused barren lands, cliffs, and agricultural lands to decrease drastically.

## 4. Discussion

The progress of industry (refinery, factories, and harbors) has provided more job opportunities. So because of proximity of industrial and commercial zones to urban regions, migration rate to Bandar Abbas has drastically increased in recent years. Statistics confirm this situation in different time intervals. There was no supervision or plan concerning settlement of migrants so marginalization increased in Bandar Abbas city. Migrants tend to settle in fringing areas as they do not earn enough money to afford settlement in urban lands and developed regions. Irregular expansion and unauthorized manipulation in rural areas are consequences of this phenomenon. Moreover, less developed regions lack environmental, economic, social, structural, and accessibility infrastructures which make settlement and security providing procedures much more difficult.

One of the most interesting characteristics of urban expansion in Bandar Abbas is that some rural areas such as Soru (West), Shahrak Shagho town (Northeast), and Nakhl-e-Nakhoda were attached to urban region. As the city was developing and lands were being occupied by urban construction and highway networks were established, the rural areas were attached to Bandar Abbas and today they are part of the city. Expansion of urban regions toward East and Northeast was resulted from construction of new infrastructures and new residential complexes. Imam Hossein Boulevard which connects Northeast to Northwest has caused higher population density during recent years. Among factors which have caused development of new settlements, development of access routes and proper urban infrastructures might be mentioned. The mentioned factors have led new migrants to tend to settle in new districts. Moreover, central regions of the city suffer from decreasing development due to old fabric which is not upgraded. Additionally, primary inhabitants of old districts in Bandar Abbas city have migrated to new regions of the city and they are replaced with new low income people. The low income people in central districts are not able to reconstruct or upgrade their settlements so decrease of development in these regions will be intensified gradually. Furthermore, urban managers and planners do not pay necessary attention to these areas which will result in irregular manipulation in empty urban lands and they dedicate more spaces to settlements and new urban uses. Expansion of urban lands toward North and Northwest is known as a crucial disadvantage of urban growth because those areas in terms of geological structure have a fragile fabric. Urban construction and ignoring coastal zone preservation rules has brought about environmental pollutions in coastal zones. Moreover, constructing a beach road in the tide region (less than 60 meters) has greatly affected tourism functionality of the city and the touristic activities are considerably decreased in the area.

## 5. Conclusions

Urban sprawl plays a significant role in positive and negative development of urban societies and land use changes. In this study, process of land use change in Bandar Abbas city during 1956–2012 is investigated and analyzed. The area of Bandar Abbas has multiplied by 12 during 60 last years. This amount of urban growth rate is scarce in Iran and even other countries of the world. This accelerated growth has led a great part of barren lands, cliffs, agricultural lands, coastal zones, and surrounding villages to join urban area. Various factors have affected urban expansion. Among them, activation of Rajaee and Bahonar harbors in the West of the city after war, migration of inhabitants of war affected regions, and establishment of refinery and steel, aluminum, and petrochemical factories might be pinpointed.

Bandar Abbas city has experienced lots of changes regarding shape and urban form. Different political, economic, social, and environmental factors have affected its formation. After war the functionality of the city changed to the most important trade, import and export center which in turn, encouraged lots of people to migrate to this city. Therefore, the demand for lands to be assigned to settlement purposes and urban construction increased. Hence, barren lands, cliffs, agricultural lands, and coastal zone were exploited for urban construction. The urban sprawl has been mostly toward East, Southeast, and Northeast. The city faces development and growth limitations from western part (because of military zone), northern region (due to cliffs, slope and railways), and southern boundary (as it reaches coastal zone). The area of Bandar Abbas city has increased fourfold during three last decades. It was 2914.64 in 1975 and it became 4959.59 hectares in 2012. The ascending migration rate has caused the population to increase sixfold. It was 87981 people in 1975 while it became 518345 in 2012. It is obvious that urban lands growth has been less than population growth which illustrates high population density in Bandar Abbas. With respect to our achievements, urban sprawl in Bandar Abbas has stimulated various land use changes in urban area. Effective factors and reasons which have led to such irregular urban sprawl should be noticed by managers and urban planners so that they could prepare a strategy for urban development of Bandar Abbas city. Some of the policies which might be utilized for control of urban sprawl are as follows: reclamation of lands inside the city and in older regions of the city to provide regional balance regarding constructions and to achieve balanced distribution of population; avoiding unauthorized construction and marginalization in outskirts; developing surrounding villages to prevent further migration; constructing residential towns; and transferring factories and industrial zones to outside of city exclusion.

## Figures and Tables

**Table 1:** Data used in this study.

Data	Pass and row	Year	Spatial resolution/scale (m)
Aerial photos			
	56-302	1956	1 : 20000
	68-438	1965	1 : 5000
	74-665	1975	1 : 15000
	88-459	1987	1 : 10000
	2793	2001	1 : 10000
Ultra CAM-d		2013	
Satellite images			
Landsat ETM+	160/41	Apr 07, 2003	15–30
IRS (panchromatic band)	75-52-D	Jul 21, 2008	5.8
GeoEye-1	—	Dec 31, 2012	0.5
Secondary data			
3D topographic map	—	2001	1 : 2000
DEM	—	2001	1 m vertical
Iranian censuses	—	1976–1986–1991–1996–2006	
UN Iran censuses	—	1995–2025	

**Table 2:** Assessment of satellite images classification.

Year	Overall accuracy (%)	Kappa coefficient
2003	97.11	0.93
2008	98.12	0.89
2012	99.86	0.98

**Table 3:** The percentage of population and area growth in Bandar Abbas city during 60 years interval.

Year	Population	Percentage increase in population (%)	Built-up area (ha)	Percentage increase in built-up area (%)	Built-up land per capita (m^2^/person)
1956	17710	—	403.77	—	22.79
1965	28434	60.55	753.78	86.69	26.50
1975	87981	209.42	1292.81	71.51	14.69
1987	174650	98.51	2914.64	125.45	16.68
2001	276578	58.36	4182.88	43.51	15.12
2012	518345	87.41	4959.59	18.57	18.93

**Table 4:** Land use classes changes during studied time periods (hectares).

	Agricultural land	Barren land	Coastal zone	Marsh land	Military land	River	Rocky hills	Urban land
1956	155.62	2536.59	210.96	4.71	48.13	162.43	2097.24	403.77
1965	133.26	2232.09	192.82	6.45	49.42	153.86	2122.15	753.78
1975	49.94	2061.80	111.52	12.64	154.86	131.90	1834.29	1292.81
1987	115.35	541.45	111.33	16.64	337.93	122.59	1511.96	2914.64
2001	30.48	237.11	101.15	9.33	340.07	109.61	661.28	4182.88
2012	6.92	47.46	60.31	8.65	296.63	72.05	262.16	4959.59

Total change	−148.70	−2489.14	−150.66	+3.94	+248.50	−90.39	−1835.08	+4555.82

**Table 5:** Land use/cover matrix, 1956–2012 (hectares).

	1956								
	Urban land	Agricultural land	Marsh land	River	Coastal zone	Military land	Rocky hills	Barren land	Total
2012									
Urban land	403.74	155.11	3.05	75.68	162.87	0.62	1782.06	2329.03	4912.17
Agricultural land	0	0	0	30.81	0	0	0	16.64	47.45
Marsh land	0	0	0	12.72	46.89	0	0	0.60	60.21
River	0	0	0	0.17	0.56	47.51	78.72	169.54	296.49
Coastal zone	0	0	0	0.00	0	0	6.92	0	6.92
Military land	0	0	0	3.04	0	0	225.04	34.06	262.14
Rocky hills	0	0.49	0	39.97	0.60	0	4.34	26.56	71.96
Barren land	0	0	1.66	0	0	0	0	6.99	8.65

Total	403.74	155.60	4.71	162.38	210.91	48.13	2097.08	2583.43	5665.99

## References

[1] United Nation World urbanization prospects: the 2007 revision. http://www.un.org/esa/population/publications/wup2007/2007WUP_Highlights_web.pdf.

[2] Alberti M (2005). The effects of urban patterns on ecosystem function. *International Regional Science Review*.

[3] Suribabu CR, Bhaskar J, Neelakantan TR (2012). Land use/cover change detection of Tiruchirapalli City, India using integrated remote sensing and GIS tools. *Journal of the Indian Society of Remote Sensing*.

[4] Li F, Liu X, Hu D (2009). Measurement indicators and an evaluation approach for assessing urban sustainable development: a case study for China's Jining City. *Landscape and Urban Planning*.

[5] Garmany J (2011). Situating Fortaleza: urban space and uneven development in northeastern Brazil. *Cities*.

[6] Catalán B, Saurí D, Serra P (2008). Urban sprawl in the Mediterranean? Patterns of growth and change in the Barcelona Metropolitan Region 1993–2000. *Landscape and Urban Planning*.

[7] Cheng J, Masser I (2003). Urban growth pattern modeling: a case study of Wuhan city, PR China. *Landscape and Urban Planning*.

[8] Gill J (2008). *The effect of urban sprawl on Sydney’s peri -urban agricultural region. Society, Environmental Policy and Sustainability [MCom thesis]*.

[9] Lata KM, Sankar Rao CH, Krishna Prasad V, Badrinath KVS, Raghavaswamy V (2001). Measuring urban sprawl: a case study of Hyderabad. *GIS Development*.

[10] Muñiz I, Calatayud D, García M, Indovina F (2007). Sprawl causes and effects of urban dispersion. *The Low Density City*.

[11] Simon E, Vidic A, Braun M, Fábián I, Tóthmérész B (2013). Trace element concentrations in soils along urbanization gradients in the city of Wien, Austria. *Environmental Science and Pollution Research*.

[12] Magura T, Lövei GL, Tóthmérész B (2010). Does urbanization decrease diversity in ground beetle (Carabidae) assemblages?. *Global Ecology and Biogeography*.

[13] Galster G, Hanson R, Ratcliffe MR, Wolman H, Coleman S, Freihage J (2001). Wrestling sprawl to the ground: defining and measuring an elusive concept. *Housing Policy Debate*.

[14] Gabriel SA, Faria JA, Moglen GE (2006). A multiobjective optimization approach to smart growth in land development. *Socio-Economic Planning Sciences*.

[15] Litman T (2007). *Evaluating Criticism of Smart Growth*.

[16] Turner MA (2007). A simple theory of smart growth and sprawl. *Journal of Urban Economics*.

[17] Weng Q (2002). Land use change analysis in the Zhujiang Delta of China using satellite remote sensing, GIS and stochastic modelling. *Journal of Environmental Management*.

[18] Jat MK, Garg PK, Khare D (2008). Monitoring and modelling of urban sprawl using remote sensing and GIS techniques. *International Journal of Applied Earth Observation and Geoinformation*.

[19] Sudhira HS, Ramachandra TV, Jagadish KS (2004). Urban sprawl: metrics, dynamics and modelling using GIS. *International Journal of Applied Earth Observation and Geoinformation*.

[20] Herold M, Goldstein NC, Clarke KC (2003). The spatiotemporal form of urban growth: measurement, analysis and modeling. *Remote Sensing of Environment*.

[21] Gennaio M-P, Hersperger AM, Bürgi M (2009). Containing urban sprawl-evaluating effectiveness of urban growth boundaries set by the Swiss land use plan. *Land Use Policy*.

[22] Haack BN, Rafter A (2006). Urban growth analysis and modeling in the Kathmandu Valley, Nepal. *Habitat International*.

[23] Yang X, Liu Z (2005). Use of satellite-derived landscape imperviousness index to characterize urban spatial growth. *Computers, Environment and Urban Systems*.

[24] Deng JS, Wang K, Hong Y, Qi JG (2009). Spatio-temporal dynamics and evolution of land use change and landscape pattern in response to rapid urbanization. *Landscape and Urban Planning*.

[25] Jothimani P Operational urban sprawl monitoring using satellite remote sensing: excerpts from the studies of Ahmedabad, Vadodara and Surat, India.

[26] Farhoudi R, Zanganeh Shahraki S, Saed Moucheshi R (2009). Spatial distribution of population in Iranian urban system. *Quarterly of Geographical Research*.

[27] Iranian Statistic Center (2012). *Census data*.

[28] Zanganeh Shahraki S, Sauri D, Serra P, Modugno S, Seifolddini F, Pourahmad A (2011). Urban sprawl pattern and land-use change detection in Yazd, Iran. *Habitat International*.

[29] Iranian Statistic Center (2009). *Census Data*.

[30] Ministry of Roads and Urban Development (2005). *Iranian Cities and Their Spatial Distribution in Different Periods*.

[31] Ministry of Roads and Urban Development (2008). *Iranian Cities and Their Spatial Distribution in Different Periods*.

[32] Serra P, Pons X, Saurí D (2003). Post-classification change detection with data from different sensors: some accuracy considerations. *International Journal of Remote Sensing*.

[33] Cunningham MA (2006). Accuracy assessment of digitized and classified land cover data for wildlife habitat. *Landscape and Urban Planning*.

[34] Barnes KB, Morgan JM, Roberge MC, Lowe S (2001). *Sprawl Development: Its Patterns, Consequences, and Measurement*.

[35] Doygun H (2009). Effects of urban sprawl on agricultural land: a case study of Kahramanmaraş, Turkey. *Environmental Monitoring and Assessment*.

[36] Epstein J, Payne K, Kramer E (2002). Techniques for mapping suburban sprawl. *Photogrammetric Engineering and Remote Sensing*.

[37] Torrens PM, Alberti M (2000). *Measuring Sprawl*.

[38] European Environment Agency (EEA) (2006). *Urban Sprawl, the Ignored Challenge*.

[39] Onur I, Maktav D, Sari M, Kemal Sönmez N (2009). Change detection of land cover and land use using remote sensing and GIS: a case study in Kemer, Turkey. *International Journal of Remote Sensing*.

[40] Akbari H, Rose LS, Taha H (2003). Analyzing the land cover of an urban environment using high-resolution orthophotos. *Landscape and Urban Planning*.

[41] Ministry of Roads and Urban Development (2012). *Iranian Cities and Their Spatial Distribution in Different Periods*.

